# Development of a ^64^Cu-labeled CD4^+^ T cell targeting PET tracer: evaluation of CD4 specificity and its potential use in collagen-induced arthritis

**DOI:** 10.1186/s13550-022-00934-7

**Published:** 2022-09-16

**Authors:** Anne Skovsbo Clausen, Camilla Christensen, Esben Christensen, Sigrid Cold, Lotte Kellemann Kristensen, Anders Elias Hansen, Andreas Kjaer

**Affiliations:** 1 Department of Clinical Physiology, Nuclear Medicine & PET and Cluster for Molecular Imaging, Department of Biomedical Sciences, Rigshospitalet and University of Copenhagen, Blegdamsvej 9, 2100 Copenhagen, Denmark; 2 Department of Health Technology, Section for Biotherapeutic Engineering and Drug Targeting, Technical University of Denmark, Anker Engelunds Vej 1, 2800 Kongens Lyngby, Denmark; 3 Minerva Imaging, Lyshøjvej 21, 3650 Ølstykke, Denmark

**Keywords:** Positron emission tomography (PET), [^64^Cu]Cu-NOTA-CD4, CD4^+^ T cells, Rheumatoid arthritis, Collagen-induced arthritis, Animal model

## Abstract

**Background:**

CD4^+^ T cells are central inflammatory mediators in the pathogenesis of autoimmune rheumatoid arthritis (RA), as they are one of the dominating cell types in synovial inflammation. Molecular imaging of CD4^+^ T cells has potential role for early detection and monitoring of RA. Here, we developed a new radiotracer for in vivo immunoPET imaging of murine CD4^+^ T cells and tested it in the collagen-induced arthritis (CIA) mouse model of human RA.

**Results:**

The tracer, [^64^Cu]Cu-NOTA-CD4-F(ab)’2 ([^64^Cu]Cu-NOTA-CD4), was generated from F(ab)’2 fragments of R-anti-mouse CD4 antibodies conjugated to the 2-*S*-(isothiocyanatbenzyl)-1,4,7-triazacyclononane-1,4,7-triacetic acid (*p*-SCN-Bn-NOTA) chelator and radiolabeled with copper-64. Accumulation of the tracer and isotype control was evaluated in the CIA model and mice receiving whole-body irradiation (WBI) (5 Gy). The potential of [^64^Cu]Cu-NOTA-CD4 for response assessment was evaluated in CIA induced mice treated with dexamethasone (DXM). Imaging data were compared with flow cytometry and immunohistochemistry (IHC) of inflammatory cells including CD4^+^ T cells. [^64^Cu]Cu-NOTA-CD4 showed increased accumulation in T cell-rich tissues compared with isotype control (*p* < 0.0001). In addition, reduced accumulation of [^64^Cu]Cu-NOTA-CD4 was observed in T cell-depleted tissue (*p* < 0.0001). Flow cytometry and IHC confirmed the increased infiltration of CD4^+^ T cells in CIA mice.

**Conclusions:**

We developed and evaluated a new radiotracer, [^64^Cu]Cu-NOTA-CD4, for immunoPET imaging of murine CD4^+^ T cells. [^64^Cu]Cu-NOTA-CD4 was successfully synthesized by F(ab)’2 fragments of R-anti-mouse CD4 antibodies conjugated to a chelator and radiolabeled with copper-64. We found that our novel CD4 PET tracer can be used for noninvasive visualization of murine CD4^+^ T cells.

**Supplementary Information:**

The online version contains supplementary material available at 10.1186/s13550-022-00934-7.

## Introduction

Rheumatoid arthritis (RA) is an autoimmune, inflammatory disease with an estimated prevalence of 1% worldwide [1]. It is a long-term, progressive disorder that can result in chronic inflammation and articular destruction of multiple joints. The pathogenesis involves T cell-mediated recognition of arthritis-specific autoantigens through major histocompatibility complex class II-mediated presentation [2, 3]. A variety of immune cells play a role in the hyperplasia progression of synovial membranes (synovium), cartilage degeneration and bone tissue destruction [4]. However, CD4^+^ T cells are key mediators of tissue damage in the joints and play a crucial role in disease initiation [5, 6]. If left undiagnosed, untreated or unresponsive to therapy, inflammation and joint destruction will lead to loss of physical function and have severe consequences for the patient including difficulties and potentially inability in maintaining daily living. Therefore, early diagnosis and treatment initiation is of paramount importance.

The diagnosis of RA mainly depends on the clinical phenotype and serology testing. However, as RA is a progressive disease, advanced disease can be assessed by imaging including X-rays, ultrasound and MRI [7]. Nevertheless, these techniques have low sensitivity of detecting articular changes at an earlier time point. The modalities are not well established in the clinic and a need for new methods for earlier detection and confirmation of cases occur. The clinical need is not limited to consistent, predictive biomarkers of prognosis, but also of therapeutic response markers in order to improve patient compliance [8]. Here, we focus on the potential advantages of using immunoPET imaging to detect the inflammatory CD4^+^ T cell response in the collagen-induced arthritis (CIA) model, a mouse model of RA. ImmunoPET has mostly been used in oncology for tumor associated antigens but has previously been expanded to several indications including imaging of infections and inflammatory diseases [9]. Using molecular imaging to target specific inflammatory biomarkers allows for visualization of cells and molecules that are involved in the process. Imaging also allows for assessment of localized inflammatory activity, which is highly relevant in RA. Finally, the early diagnosis and treatment initiation of patients with inflammatory conditions is crucial, as it can prevent irreversible tissue damage.

As RA is a T cell-mediated disease, we hypothesized that application of murine T cell-specific antibody F(ab)′2 fragments could be useful to track T cell migration in the CIA model [10]. In the present study, we therefore developed a new radiotracer for noninvasive immunoPET of murine CD4^+^ T cells. F(ab)′2 fragment targeting murine CD4^+^ T cells was conjugated to the chelator 2-*S*-(isothiocyanatbenzyl)-1,4,7-triazacyclononane-1,4,7-triacetic acid (*p*-SCN-Bn-NOTA) and radiolabeled with copper-64. [^64^Cu]Cu-NOTA-CD4-F(ab)’2 ([^64^Cu]Cu-NOTA-CD4) was evaluated as a predictive imaging biomarker in the CIA model. First, the optimal imaging time point was determined in a longitudinal biodistribution study. Next, the uptake of [^64^Cu]Cu-NOTA-CD4 following systemic administration of dexamethasone (DXM) was assessed in inflamed arthritic lesions. Finally, target-specific affinity of [^64^Cu]Cu-NOTA-CD4 and the isotype control radiotracer [^64^Cu]Cu-NOTA-IgG2b-F(ab)’2 ([^64^Cu]Cu-NOTA-IgG2b) was evaluated. To validate the findings using other methods, infiltration of CD4^+^ T cells in the inflamed joints was assessed by flow cytometry and immunohistochemistry (IHC) and compared with CD4 PET imaging.

## Materials and methods

### Mice

Female DBA/1JRj mice (7 weeks) and C57BL/6JRj (7 weeks) were purchased (Janvier, Le Genest-Saint-Isle, France) and housed in groups of 4–8 mice in individually ventilated cages under standardized lighting conditions. Mice were fed pathogen-free food and water ad libitum. All animal experiments were conducted under protocols approved by the National Animal Experiments Inspectorate under the license number 2016-15-0201-00920.

### Collagen-induced arthritis (CIA)

Mice were immunized subcutaneously at the base of the tail (100 µL) with chicken CII (Chondrex, Redmond, WA, USA) emulsified in complete Freund's adjuvant (Sigma-Aldrich, St. Louis, MO, USA). Three weeks after initial immunization, mice received a booster immunization with CII emulsified in incomplete Freund's adjuvant (Sigma-Aldrich, St. Louis, MO, USA) (100 µL) [11]. CII was dissolved O/N at 4 °C in 10 mM acetic acid. Mice were anesthetized in 3–4% sevoflurane in 65% N_2_ and 35% O_2_ during both injections. Signs of arthritis in the paws appeared approximately four weeks after initial immunization. Each paw was visually scored three times a week on a scale from 0 to 4. The following criteria were used: 0: normal paw, 1: one toe inflamed and swollen, 2: more than one toe, but not entire paw, inflamed and swollen or mild swelling of entire paw, 3: entire paw inflamed and swollen and 4: very inflamed and swollen paw or ankylosed paw [12]. For ethical purposes, a mean score of 2.5 for all paws was considered maximum per animal. Mice were euthanized if exceeding the mean score. Data are presented as the mean score of all four paws. In case of lameness, score ≥ 2, the mice were treated with daily analgesia (buprenorphine, 1 mg/kg, intraperitoneal (i.p.)). For therapeutic assessment, the CIA mice were treated daily with DXM for 7 days starting on day 28 after initial immunization [13–15]. DXM was administered as i.p. injections in a total volume of 200 µL/mouse (1 mg/kg in NaCl).

### Radiosynthesis of [^64^Cu]Cu-NOTA-CD4 and isotype control

F(ab)’2 fragments were generated from the GK1.5 monoclonal antibody R-anti-mouse CD4 (#BP0003-1, Bio X cell, Lebanon, NH, USA) and R-anti-mouse IgG2b clone LTF-2 (#BE0090, Bio X cell) using FabRICATOR enzyme (#A0-FR1-050, Genovis, Lund, Sweden). 2.5 mg CD4-F(ab)′2 and IgG2b-F(ab)’2 in PBS was incubated with 500 units FabRICATOR for 2.5 h at 37 °C. The crude antibody–enzyme mixture was purified by preparative HPLC (Yarra-2000 SEC column, 0.1 M phosphate buffer, 1 ml/min) yielding isolated F(ab)’2 and Fc fragments. The fragments purified followed by conjugation to the 2-*S*-(isothiocyanatbenzyl)-1,4,7-triazacyclononane-1,4,7-triacetic acid (*p*-SCN-Bn-NOTA) chelator, as previously described [16].

[^64^Cu]Cl_2_ (DTU Nutech, DTU) was dissolved in TraceSelect water (Merck Millipore) to a final concentration of 2 GBq/mL. NOTA-CD4-F(ab)’2 (100 µg in PBS) was incubated with 250 MBq [^64^Cu]Cl_2_ in 0.1 M NaoAc buffer pH = 5.5 with 5 mg/ml gentisic acid (15 min, 37 °C). The reaction was quenched with 10 mM EDTA followed by PD10 purification into PBS. The radiochemical yield and purity at end-of-synthesis (EOS) were determined by size-exclusion-chromatography-HPLC (SEC-HPLC) using an isocratic method with 0.1 M phosphate buffer pH = 7 as mobile phase and a flow rate of 1 ml/min.

### SDS-PAGE

Full-length monoclonal CD4 antibody (IgG), reduced full-length CD4 antibody and the tracer [^64^Cu]Cu-NOTA-CD4 were mixed with NuPAGE LDS sample buffer (#NP0007, Invitrogen, Carlsbad, CA, USA) and denatured for 10 min at 70 °C and loaded onto BoltTM 4–12% Bis–Tris gels (#NW04120, Invitrogen). Electrophoresis was run on the Mini Gel Tank (Thermo Fisher Scientific, Waltham, MA, USA) at 200 V constant voltage in NuPAGE MES SDS running buffer (#NP0002, Invitrogen). The SDS-PAGE gel was fixed and stained using Coomassie brilliant blue R-250 (#1610436, Bio-Rad, Hercules, CA, USA). The gel was analyzed for radioactive content by exposure to Multisensitive Phosphor Screens and imaged using the Amersham Typhoon Imaging System (GE Healthcare, Chicago, IL, USA).

### Mouse T cell isolation, immunoreactivity and saturation binding assay

Murine CD4^+^ cells were isolated from mouse spleen using the magnetic activating cell sorting (MACS) technique (CD4^+^ T cell isolation kit, #130-104-454; CD8a^+^ T cell isolation kit, #130-104-075, Miltenyi Biotec). Female DBA/1JRj mice (7 weeks old) were killed by cervical dislocation and the spleens removed aseptically.

The immunoreactivity of anti-CD4-F(ab)’2 fragments following radiolabeling was assessed according to the Lindmo assay [17]. Increasing concentrations of CD4^+^cells (2.5 × 10^6^–4 × 10^7^ cells/mL) were incubated with 1 nM [^64^Cu]Cu-NOTA-CD4 for 3 h at 4 °C. Cells were centrifuged at 500 g for 5 min and the supernatants and pellets counted in a gamma counter (Wizard^2^, PerkinElmer, Massachusetts, USA). Cell-associated radioactivity was calculated as the ratio of cell-bound radioactivity to the total amount of added radioactivity.

The affinity of radiolabeled anti-CD4-F(ab)’2 was assessed by a saturation binding assay. T cells were harvested as described above, added in triplicates (2 × 10^4^ cells) to a MultiScreenHTS BV Filter Plate 1.2 µm (#MSBVN1250, Merck Millipore) and washed twice in PBS. Eight different concentrations of [^64^Cu]Cu-NOTA-CD4 [range: 60–0.04 nM] in PBS supplemented with 1% bovine serum albumin (BSA) were added the wells. The plate was incubated for 4 h at 4 °C. After incubation, the plate was washed 3 times in PBS with 1% BSA using a vacuum manifold (Macherey-Nagel, Fisher Scientific). The plastic cover was removed from the plate bottom and the plate dried in a heat cabinet. The dry filters were transferred to counting tubes and counted in a gamma counter.

### In vivo PET/CT imaging

Mice were intravenous (i.v.) injected with [^64^Cu]Cu-NOTA-CD4 and 30 µg CD4-F(ab)’2 co-dose in a total volume of 150 µL (diluted in PBS) in the tail vein as a single bolus. Co-doses was given to decrease binding to lymphoid tissue significantly, but without blocking the specific uptake. The co-dose was based on previously reported data where the optimal dose was found to be 30 µg [16]. Whole-body imaging consisted of a CT acquisition followed by a static PET acquisition and was performed on the Inveon® small animal imaging system (Siemens Medical Systems, Malvern, PA, USA). Mice were anesthetized in 3–4% sevoflurane in 65% N_2_ and 35% O_2_ prior to imaging. All CIA mice were injected with tracer on day 35 after initial immunization. For longitudinal imaging evaluation, the PET/CT imaging was performed 1, 4, 24 and 44 h post-injection (p.i.) of [^64^Cu]Cu-NOTA-CD4 (2.52 ± 0.04 MBq, 0.5 ± 0.02 µg) and 30 µg CD4-F(ab)’2 co-dose in CIA (*n* = 6) and control mice (*n* = 4). For evaluation of DXM, PET/CT imaging was performed 24 h p.i. on day 36 after initial immunization of CIA (*n* = 23) and DXM-treated mice (*n* = 12) (four independent experiments: EXP1-4). Untreated control mice (*n* = 11) were also included. All mice received [^64^Cu]Cu-NOTA-CD4 (EXP1: 1.26 ± 0.02 MBq, 0.4 ± 0.02 µg; EXP2: 7.51 ± 0.29 MBq, 0.55 ± 0.03 µg; EXP3: 1.86 ± 0.10 MBq, 0.38 ± 0.01 µg; EXP4: 2.33 ± 0.02 MBq, 0.41 ± 0.01 µg) and 30 µg CD4-F(ab)’2 co-dose. For isotype control imaging, CIA (*n* = 8) and control mice (*n* = 6) were i.v. injected with [^64^Cu]Cu-NOTA-IgG2b, an isotype-matched non-specific control IgG antibody fragment labeled with ^64^Cu (1.95 ± 0.04 MBq, 0.39 ± 0.01 µg) and IgG2b-F(ab)’2 30 µg co-dose in a total volume of 150 µL (diluted PBS) in the tail vein as a single bolus. The mice were subjected to PET/CT imaging with 900 s static PET acquisition 24 h p.i. according to the previous described protocol. Whole-body irradiation (WBI) (*n* = 8/group) and non-irradiated control mice (*n* = 6/group) were either injected with [^64^Cu]Cu-NOTA-CD4 (2.40 ± 0.03 MBq, 0.60 ± 0.01 µg) or [^64^Cu]Cu-NOTA-IgG2b (2.25 ± 0.04 MBq, 0.56 ± 0.01 µg) and 30 µg co-dose. PET data were acquired in list mode with an acquisition time of 300, 300, 600 and 1200 s for the 1, 4, 24 and 44 h time points, respectively. PET scans were reconstructed using a maximum a posteriori algorithm with CT-based attenuation correction.

### Imaging analysis

Image analysis was performed using the Inveon® Research Workstation software (Siemens Medical Systems, PA, USA). A CT-based region of interest (ROI) tool was used to carefully draw around each carpal and tarsal joint, forming four ROIs per mouse. ROIs were also drawn over the heart, kidney, spleen, thymus, liver and thigh muscle. The activity in the blood was calculated as 20% maximum activity in the heart. The uptake of [^64^Cu]Cu-NOTA-CD4 and [^64^Cu]Cu-NOTA-IgG2b was quantified as percent injected dose per joint (%ID/joint) and maximum percent injected dose per gram tissue (%Max ID/g) assuming a soft tissue density of 1 g/cm^3^. Target-to-blood ratios of [^64^Cu]Cu-NOTA-CD4 uptake were calculated as maximum uptake (%Max ID/g) in the most affected joints (score 3) divided by mean uptake in the blood (%ID/g) to determine optimal scanning time. Moreover, joint-to-blood ratios were calculated for the DXM assessment.

### Whole-body irradiation (WBI)

WBI was performed to test the effect of irradiation on CD4^+^ T cells in spleen for evaluation of tracer specificity. Mice were exposed to a single γ-radiation dose of 5 Gy, 1 Gy/min (320 kV, 12.5 mA) using an X-RAD 320 (PXi, North Branford, CT, USA). Two groups of C57BL/6JRj mice (20 weeks) received WBI the day before injection of [^64^Cu]Cu-NOTA-CD4 (*n* = 8) or [^64^Cu]Cu-NOTA-IgG2b (*n* = 8). Two groups of healthy non-irradiated C57BL/6 control mice (*n* = 6/group) were also included.

### Immunohistochemical analysis

CIA and control mice were euthanized by cervical dislocation and the carpal and tarsal joints were isolated. Joints were decalcified for three weeks in 10% formic acid in buffered formaldehyde 4% followed by preparation in Shandon Excelsior AS Tissue Processor (Thermo Fisher Scientific, Waltham, MA, USA) O/N and embedded in paraffin. Paraffin-embedded joints were sectioned at 4 µM and dewaxed through xylene to tap water. For antigen retrieval, sections were boiled in microwave for 15 min in 10 mmol citrate buffer (pH 6) and pre-incubated in 2% bovine serum albumin (BSA) for 10 min followed by incubation with primary recombinant anti-CD4 antibody (#ab183685, Abcam, Cambridge, UK) at 1:500 dilution in 2% BSA O/N at 4 °C. Sections were incubated for 40 min with biotinylated secondary goat-anti-rabbit IgG antibody (BA-1000, Vector Laboratories, Burlingame, CA, USA) at 1:200 dilution. Afterward, 3% hydrogen peroxide blocked the endogenous peroxidase. To amplify the reaction, sections were incubated with Avidin and Biotinylated horseradish peroxidase macromolecular Complex (ABC-Elite) (PK-6100, Vector Laboratories) for 30 min. Finally, the reaction was developed by the use of 3,3–diaminobenzidine (SK-4100, Vector Laboratories) for 15 min and counterstaining was performed with Mayer’s Hematoxylin Solution (Sigma-Aldrich, St. Louis, MO, USA). All procedures were performed at RT if not stated otherwise. Sections were stained in the same analysis. Images were taken using an Olympus BX51 microscope with a XC-10 camera.

### Flow cytometry

Joint-infiltrating cells were isolated by a procedure adapted from [18]. Briefly, the carpal and tarsal joints were isolated. Skin and fur were removed, and joints were mechanically dissociated using surgical scissors followed by digestion in RPMI 1640 supplemented with type 1-S hyaluronidase from bovine testes (2.4 mg/mL) (Sigma, St. Louis, MO, USA), collagenase VIII (1 mg/mL) (Sigma), 10 mM HEPES, 50 units/mL penicillin and 50 µg/mL streptomycin for 1 h at 37 °C in a shaking water bath. Digested joints were passed through a 70 µm cell strainer.

Processed cells were blocked with Fc-block (clone 2.4G2) (BD Biosciences, San Jose, CA, USA) in FACS buffer (PBS + 0.5% BSA + 0.1% NaN3 + 2 mM EDTA) for 5 min on ice. Fc-blocked samples were stained for 30 min on ice in a master mix of FACS buffer, brilliant stain buffer, amine reactive dye (eFluor780 viability dye) (Thermo Fisher Scientific, Waltham, MA, USA) and the following antibodies: anti-CD3 (clone 145-2C11) PE-CF594, anti-CD4 (clone RM4-5) BV786, anti-CD8 (clone 53–6.7) APC, anti-CD11b (clone M1/70) BV480, anti-CD11c (clone N418) PE-Cy7, anti-CD45 (clone 30-F11) AF700, anti-CD64 (clone X54-5/7.1) BV421, anti-I-A/I-E (clone M5/114.15.2) and anti-Ly6g (clone 1A8) BV711. Antibodies against CD3, CD4, CD8, CD11b, CD45, CD64 and Ly6g were purchased from BD Biosciences. Antibodies against CD11c and I-A/I-E were purchased from BioLegend (BioLegend, San Diego, CA, USA). Cells were acquired on a BD LSRFortessa X-20 using BD FACSDiva v.8.0.1 software (BD Biosciences, San Jose, CA, USA). Data analysis was performed in FlowJo v.10.6.1, Tree Star Inc. (FlowJo, Ashland, OR, USA).

### Statistical analysis

One-way analysis of variance with Tukey’s post hoc test was used to assess statistically significant differences between groups. *p* values < 0.05 were considered statistically significant. Prism 8.0c (GraphPad Software, La Jolla, CA, USA) was used for all statistical analysis.

## Results

### Radiosynthesis of [^64^Cu]Cu-NOTA-CD4, immunoreactivity and saturation binding assay

**Fig. 1:**
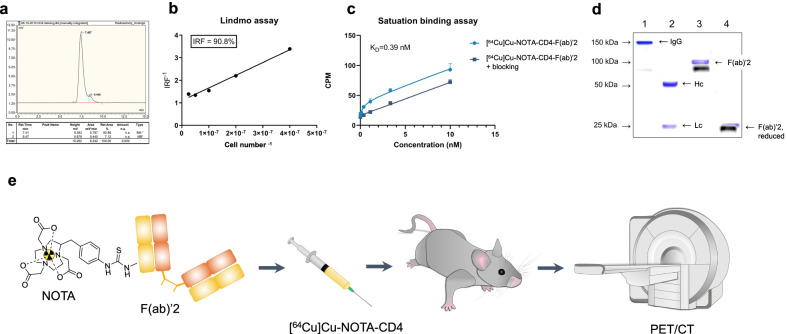
Development of [^64^Cu]Cu-NOTA-CD4: a CD4^+^ T cell targeting PET tracer. **a** Representative radio-HPLC chromatogram of [^64^Cu]Cu-NOTA-CD4. **b** Immunoreactivity of [^64^Cu]Cu-NOTA-CD4. **c** K_D_ value of [^64^Cu]Cu-NOTA-CD4. **d** Merged and cropped SDS-PAGE of Coomassie staining and radiography of the same gel SDS-PAGE gel. Lane 1: Full-length anti-mouse CD4 antibody (IgG); Lane 2: Full-length anti-mouse CD4 antibody, reduced. Hc: Heavy chain. Lc: Light chain; Lane 3: [^64^Cu]Cu-NOTA-CD4; and Lane 4: [^64^Cu]Cu-NOTA-CD4, reduced. **e** The cyclic chelator NOTA was conjugated to F(ab)’2 fragments of R-anti-mouse CD4 antibodies and radiolabeled with Cu-64. Mice were i.v. injected with [^64^Cu]Cu-NOTA-CD4 and co-dose prior to PET/CT imaging

The F(ab)’2 fragments of R-anti-mouse CD4 antibodies were successfully conjugated to NOTA and radiolabeled with Cu-64 with a radiochemical yield of 79.5 ± 1.5% and a specific activity of 6.5 ± 3.7 MBq/µg. The produced tracer [^64^Cu]Cu-NOTA-CD4 had a high radiochemical purity (> 90%) and aggregates estimated to < 5% by SEC-HPLC. Representative radiochromatogram of [^64^Cu]Cu-NOTA-CD4 is presented in Fig. 1a. The immunoreactivity of [^64^Cu]Cu-NOTA-CD4 toward CD4 expressing splenocytes was estimated to 90.8% (Fig. 1b). [^64^Cu]Cu-NOTA-CD4 exhibited affinity toward CD4 expressing solenocytes in the nanomolar range with an estimated K_D_ of 0.39 nM (Fig. 1c).

SDS-PAGE analysis by Coomassie staining and radiography of the same gel, confirmed the digestion efficiency and revealed [^64^Cu]Cu-NOTA-CD4-F(ab)’2 at 100 kDa (lane 3) with no presence of digested fragments or full-length antibody in the final product. In addition, the full-length anti-mouse CD4 antibody (IgG) at 150 kDa (lane 1), the digested antibody (lane 2) with heavy chains (Hc) and light chains (Lc) are shown (Fig. 1d). The full-length SDS-PAGE stained is shown in Additional file 1: Fig S1a, and radiography of the full-length gel (only exposed once) is shown in Additional file 1: Fig. S1b.

### Longitudinal PET evaluation of [^64^Cu]Cu-NOTA-CD4 in arthritic lesions

**Fig. 2:**
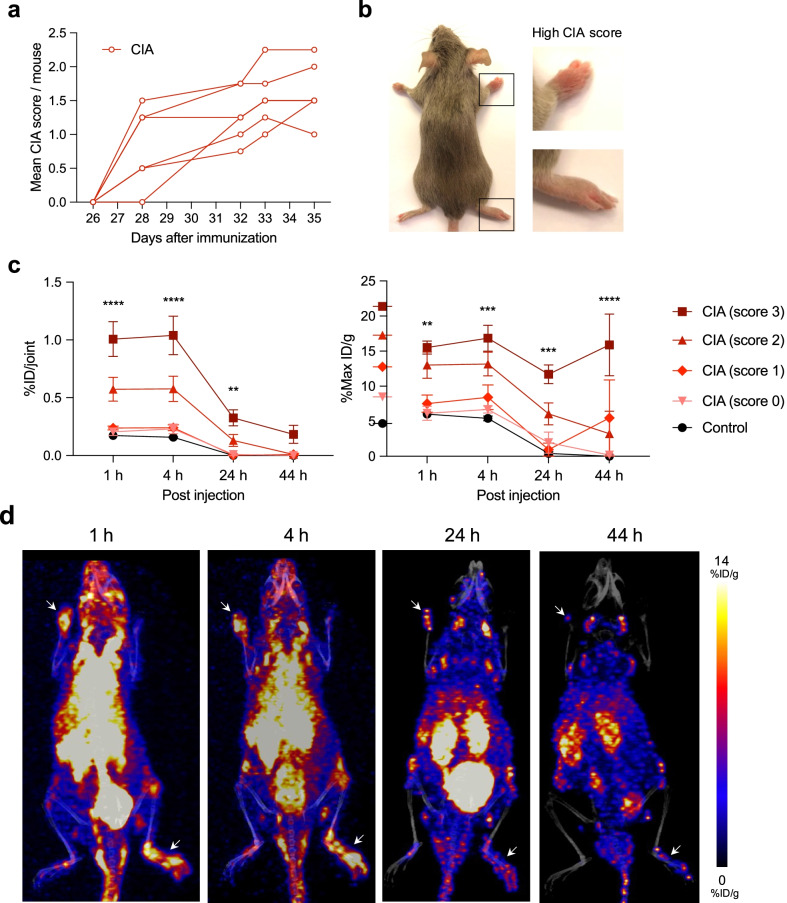
Characterization of the collagen-induced arthritis (CIA) mouse model and [^64^Cu]Cu-NOTA-CD4 uptake in joints. **a** CIA mice were scored according to degree of inflammation (swelling and erythema) in each paw on a scale from 0 to 4 *(n* = 6)*.* Data are shown as mean CIA score per mouse starting on day 26 after immunization to the last day of PET/CT imaging where the mice were euthanized (day 35). Each line represents the development of arthritis in one mouse. **b** Representative images of a CIA mouse on day 35 with pronounced arthritis in right carpal and tarsal joints. **c** [^64^Cu]Cu-NOTA-CD4 uptake per joint (%ID/joint) and maximum activity (%Max ID/g) in carpal and tarsal joints of CIA mice (*n* = 6) and control mice (*n* = 4) at 1, 4, 24 and 44 h p.i. Asterisks (*) represent statistical significance between controls and mice with high CIA score (score 3). Data are presented as mean ± SEM. (*). *p* < 0.01 (**), *p* < 0.001 (***) and *p* < 0.0001 (****). **d** Representative maximum intensity projections (coronal view) of a CIA mouse with pronounced arthritis (score 3) in left carpal and right tarsal joint at 1, 4, 24 and 44 h p.i. of [^64^Cu]Cu-NOTA-CD4. White arrows indicate lesion sites with score 3

Mice immunized with chicken CII emulsion in CFA (day 0) followed by a booster immunization with the emulsion in IFA (day 21) developed macroscopic joint inflammatory including swelling and erythema of carpal and tarsal joints starting on day 28 after initial immunization. Monitoring of animals was performed on day 28, 32, 33 and 35 with mean CIA score per animal of 0.83 ± 0.24, 1.29 ± 0.16, 1.54 ± 0.18 and 1.63 ± 0.18, respectively (Fig. 2a, b).

The in vivo accumulation of [^64^Cu]Cu-NOTA-CD4 was studied in a longitudinal PET evaluation of CIA mice 1, 4, 24 and 44 h p.i. [^64^Cu]Cu-NOTA-CD4 displayed a significantly increased activity in the most affected joints (score 3) 1 h (1.01 ± 0.15% ID/joint, *p* < 0.0001), 4 h (1.04 ± 0.17% ID/joint, *p* < 0.0001) and 24 h (0.33 ± 0.07% ID/joint, *p* = 0.0052) p.i. in comparison with control mice. The uptake in the most pronounced arthritic joints had a drop after the two initial time points in comparison with the two latter time points. At the 44 h p.i. imaging time point the activity was 0.18 ± 0.08% ID/joint. Moreover, the maximum uptake within the most affected joints (score 3) was 15.5 ± 0.9, 16.8 ± 1.8, 11.7 ± 1.3 and 15.9 ± 4.4% ID/g for the 1, 4, 24 and 44 h time points, respectively. The maximum uptake in joints with score 3 was significantly increased at all time points compared with control mice (1 h: *p* = 0.0079; 4 h: *p* = 0.0003; 24 h: *p* = 0.0003 and 44 h: *p* < 0.0001) (Fig. 2c).

Representative PET/CT images of a CIA mouse at 1, 4, 24 and 44 h p.i. of [^64^Cu]Cu-NOTA-CD4 are shown in Fig. 2d. The presented mouse had obvious clinical symptoms of arthritis including swelling and erythema of the left carpal and right tarsal joint, which mutually scored 3. The increased activity in these arthritic joints is visualized at all time points. Clearance of the background can also be seen throughout the temporal imaging.

**Fig. 3:**
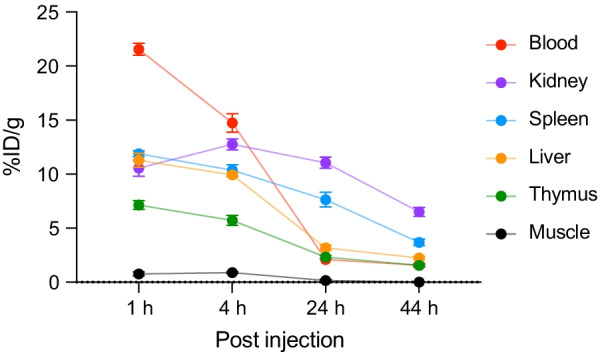
In vivo biodistribution of [^64^Cu]Cu-NOTA-CD4. [^64^Cu]Cu-NOTA-CD4 accumulation in selected organs: blood, kidney, spleen, liver, thymus and muscle of collagen-induced arthritis mice expressed as %ID/g. Images were acquired 1, 4, 24 and 44 h p.i. (*n* = 6). All data are presented as mean ± SEM

In vivo biodistribution profile of [^64^Cu]Cu-NOTA-CD4 was performed in the organs of interest. The organ of interest was spleen and thymus, since they are lymphoid organs, blood for circulation time and liver and kidney for excretion. The organ of interest showed high accumulation in blood, kidneys, spleen, liver and thymus at the initial time points (1 and 4 h) followed by a decrease at the late time points (24 and 44 h) (Fig. 3). The activity in the blood measured as 20% maximum activity in ROIs drawn over the heart decreased between the initial and late time point. Specifically, blood activity decreased from 21.6 ± 0.5 and 14.7 ± 0.9% ID/g (1 and 4 h p.i.) to 2.1 ± 0.3 and 1.6 ± 0.2% ID/g (24 and 44 h p.i.). This demonstrates the renal and hepatic clearance of tracer from blood.

Joint-to-blood ratios of [^64^Cu]Cu-NOTA-CD4 uptake in most affected joints (score 3) significantly increased throughout the imaging time points (1 to 44 h) (*p* = 0.0071) (Table 1). Based on these results, 24 h p.i. was chosen as PET imaging time point for the therapy assessment. This was chosen although the highest joint-to-blood ratio of [^64^Cu]Cu-NOTA-CD4 was detected 44 h p.i.

**Table 1:** Joint-to-blood ratios of [^64^Cu]Cu-NOTA-CD4 uptake in joints of collagen-induced arthritis mice with high arthritis score (score 3) (*n* = 13/time point). Ratios are presented as mean ± SEM

	Joint/blood
1 h	1.69 ± 0.05
4 h	1.17 ± 0.11
24 h	5.57 ± 1.31
44 h	10.45 ± 2.40

### [^64^Cu]Cu-NOTA-CD4 accumulation correlates with arthritic inflammation levels in mice

**Fig. 4:**
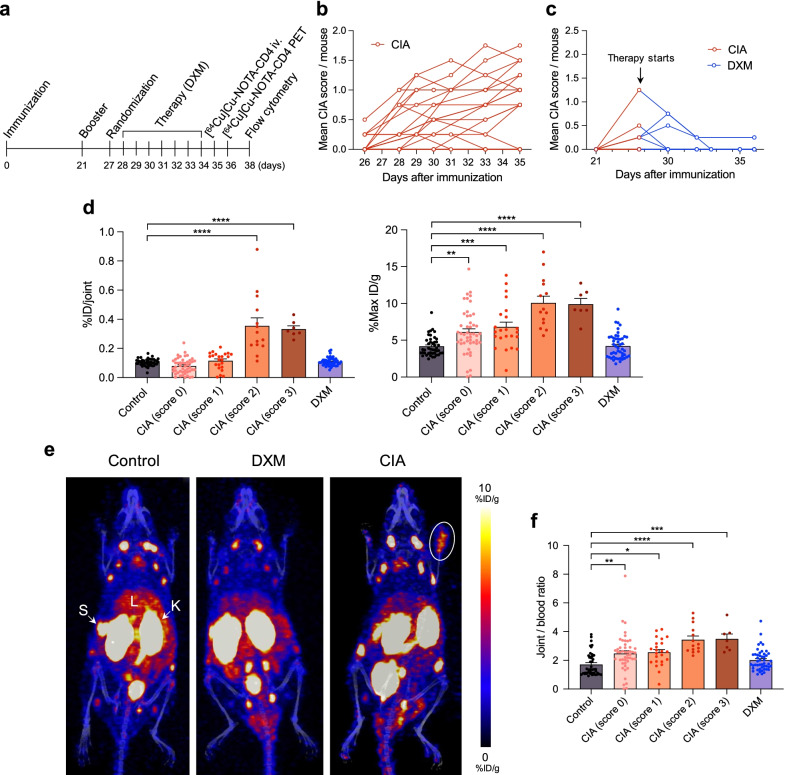
Therapy assessment and quantitative analysis of [^64^Cu]Cu-NOTA-CD4 in arthritic lesions 24 h p.i. **a** Timeline of the overall experiment starting at with immunization of collagen-induced arthritis (CIA) mice followed by booster injection three weeks later, treatment regimen, i.v. injection of [^64^Cu]Cu-NOTA-CD4 and 24 h PET/CT imaging. Flow cytometric analysis of the carpal and tarsal joints was performed on the last day of the experiments. **b** Untreated CIA mice were scored according to degree of inflammation (swelling and erythema) in each paw on a scale from 0 to 4 *(n* = 23)*.* Data are shown as mean CIA score per mouse starting on day 26 after immunization to injection of tracer (day 35). Each line represents the development of arthritis in one mouse. **c** Mean CIA score per mouse of dexamethasone (DXM) (1 mg/kg, i.p.)-treated CIA mice (*n* = 12). **d** Quantitative analysis of [^64^Cu]Cu-NOTA-CD4 uptake 24 h p.i. in the carpal and tarsal joints of control (*n* = 11), DXM treated (*n* = 12) and untreated CIA mice (*n* = 23). Uptake is calculated as %ID/joint and %Max ID/g. Each point represents a joint. **e** Representative maximum intensity projections (coronal view) of control, DXM treated and untreated CIA mice. Images were acquired 24 h p.i. as maximum intensity projections (%ID/g). Liver (L), spleen (S), kidney (K) and white ring encircle a joint with high [^64^Cu]Cu-NOTA-CD4 activity. **f** Joint/blood ratios of [^64^Cu]Cu-NOTA-CD4 uptake in control, DXM treated and untreated CIA mice 24 h p.i. Data are presented as mean ± SEM. The significance level is indicated by asterisks (*). *p* < 0.05 (*), *p* < 0.01 (**), *p* < 0.001 (***) and *p* < 0.0001 (****)

DXM, a corticosteroid with known anti-inflammatory effects, was used to evaluate the potential of [^64^Cu]Cu-NOTA-CD4 to differentiate between changes in CD4^+^ T cells in both systemically DXM treated and untreated arthritic lesions. The study was performed according to the setup in Fig. 4a. The mice were monitored on day 26, 28, 29, 30, 31, 33 and 35, and the mean CIA score for mice with arthritic lesions was 0.10 ± 0.04, 0.41 ± 0.12, 0.75 ± 0.16, 0.55 ± 0.09, 0.88 ± 0.18, 0.80 ± 0.11 and 0.93 ± 0.12, respectively (Fig. 4b). Treatment was initiated on day 28 after initial immunization. At this time point, 5 out of 12 mice (41.7%) showed initial signs of disease with a mean CIA score of 0.21 ± 0.11. Mice displayed an overall decrease in CIA score after receiving DXM (Fig. 4c).

The in vivo [^64^Cu]Cu-NOTA-CD4 PET activity was quantified 24 h p.i. in DXM and untreated CIA mice. [^64^Cu]Cu-NOTA-CD4 uptake in the most affected joints (score 3: 0.33 ± 0.02%ID/joint and score 2: 0.35 ± 0.06% ID/joint) was significantly increased in comparison with controls and DXM (0.01 ± 0.00 and 0.11 ± 0.00% ID/joint, respectively) (*p* < 0.0001). The maximum activity within the joints was 9.89 ± 0.78, 10.07 ± 0.93, 6.82 ± 0.65 and 6.10 ± 0.44%ID/g of the CIA mice with score 3, 2, 1 and 0, respectively. Compared with controls and DXM (4.17 ± 0.20% ID/g and 4.21 ± 0.24% ID/g), the maximum activity was significantly increased in all CIA mice (score 3 and 2: *p* < 0.0001; score 1: *p* = 0.0005; score 0: *p* = 0.0023) (Fig. 4d). Representative PET/CT images of a control, DXM and an untreated CIA mouse are presented in Fig. 4e. PET images showed tracer uptake in kidneys, spleens and to a lesser extent in the livers, consistent with previous biodistribution results. Furthermore, PET images demonstrated increased [^64^Cu]Cu-NOTA-CD4 uptake in the affected joints compared with control, DXM and unaffected joints (score 0). The CIA mouse shown had score 3 of the right carpus. Joint-to-blood ratios of [^64^Cu]Cu-NOTA-CD4 uptake 24 h p.i. were significantly higher in CIA mice compared with control (score 3: *p* = 0.0002; score 2: *p* < 0.0001; score 1: *p* = 0.012; score 0: *p* = 0.0031) and DXM-treated mice (score 3: *p* = 0.0032; score 2: *p* < 0.0001; score 1 and 0: *ns*) (Fig. 4f). Asterisks indicating significance levels of DXM-treated mice are not shown on graph.

### [^64^Cu]Cu-NOTA-CD4 and isotype control accumulation in arthritic joints and T cell-rich tissues

**Fig. 5:**
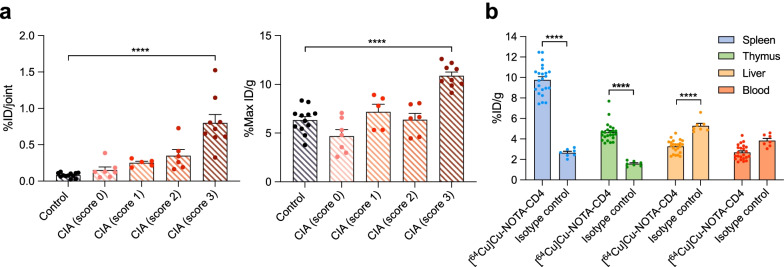
Isotype control uptake on PET images of collagen-induced arthritis (CIA) mice. **a** [^64^Cu]Cu-NOTA-IgG2b imaging 24 h p.i. of the carpal and tarsal joints of control (*n* = 6) and CIA mice (*n* = 8). Tracer accumulation is calculated as %ID/joint and %Max ID/g. Each point represents a joint. **b** Comparison of [^64^Cu]Cu-NOTA-CD4 (*n* = 23) and isotype control, [^64^Cu]Cu-NOTA-IgG2b (*n* = 7) uptake in spleen, thymus, liver and blood of CIA mice expressed as %ID/g. Data are presented as mean ± SEM. The significance level is indicated by asterisks (*). *p* < 0.0001 (****)

For evaluation of in vivo specific targeting of [^64^Cu]Cu-NOTA-CD4, a control isotype imaging study was conducted in CIA mice. This was performed to determine if the activity in inflamed joints was due to interaction between [^64^Cu]Cu-NOTA-CD4 and CD4^+^ T cells. The in vivo [^64^Cu]Cu-NOTA-IgG2b uptake was quantified 24 h p.i. as previous. Results revealed increased [^64^Cu]Cu-NOTA-IgG2b activity in joints with high arthritis score (score 3: 0.80 ± 0.12% ID/joint and 10.88 ± 0.39% Max ID/g) in comparison with control mice (0.15 ± 0.04% ID/joint and 4.69 ± 0.66% Max ID/g) (*p* < 0.0001) (Fig. 5a).

To distinguish specific from non-specific binding in relevant organs, in vivo biodistribution of the isotype was performed and [^64^Cu]Cu-NOTA-IgG2b accumulation in the spleen (2.65 ± 0.15% ID/g), thymus (1.58 ± 0.09% ID/g), liver (5.28 ± 0.24% ID/g) and blood (3.84 ± 0.22% ID/g) was calculated. A comparison between [^64^Cu]Cu-NOTA-CD4 and the [^64^Cu]Cu-NOTA-IgG2b showed significantly increased activity of [^64^Cu]Cu-NOTA-CD4 in the spleen (9.77 ± 0.31% ID/g) and thymus (4.65 ± 0.19% ID/g) (*p* < 0.0001). In addition, isotype activity was significantly increased in the liver (3.28 ± 0.13% ID/g) compared with [^64^Cu]Cu-NOTA-CD4 (*p* < 0.0001) (Fig. 5b).

### Lymphodepleting whole-body irradiation reduces [^64^Cu]Cu-NOTA-CD4 but not isotype control activity in the spleen

**Fig. 6:**
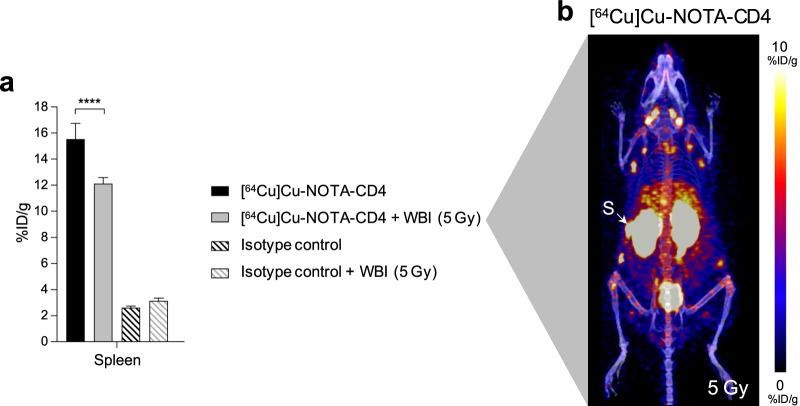
[^64^Cu]Cu-NOTA-CD4 and [^64^Cu]Cu-NOTA-IgG2b PET imaging of C57BL/6 mice after whole-body irradiation (WBI) of 5 Gy. **a** [^64^Cu]Cu-NOTA-CD4 and ^64^Cu-isotype control uptake in the spleen of WBI (5 Gy) (*n* = 8/group) and non-irradiated control mice (*n* = 6/group) expressed as %ID/g. Images were acquired 24 h p.i. (48 h after irradiation). Spleen (S). Data are presented as mean ± SEM. The significance level is indicated by asterisks (*). *p* < 0.0001 (****). **b** Representative maximum intensity projection (coronal view) of a WBI mouse 24 h p.i. of [^64^Cu]Cu-NOTA-CD4, illustrating uptake in spleen (S)

Due to the high activity of [^64^Cu]Cu-NOTA-CD4 in the spleen and thymus compared with [^64^Cu]Cu-NOTA-IgG2b, a biodistribution study in WBI and non-irradiated C57BL/6 mice was conducted to test for tracer specificity to CD4^+^ T cells. A significantly lower accumulation of [^64^Cu]Cu-NOTA-CD4 was observed in WBI (12.12 ± 1.20% ID/g) compared with non-irradiated control mice (15.54 ± 0.46% ID/g) (*p* < 0.0001). This indicates a depletion of CD4^+^ cells in the spleen following a radiation dose of 5 Gy (Fig. 6a). A representative [^64^Cu]Cu-NOTA-CD4 PET/CT image of a WBI mouse is displayed in Fig. 6b.

### Increased levels of CD4^+^ T cells in arthritic lesions

**Fig. 7:**
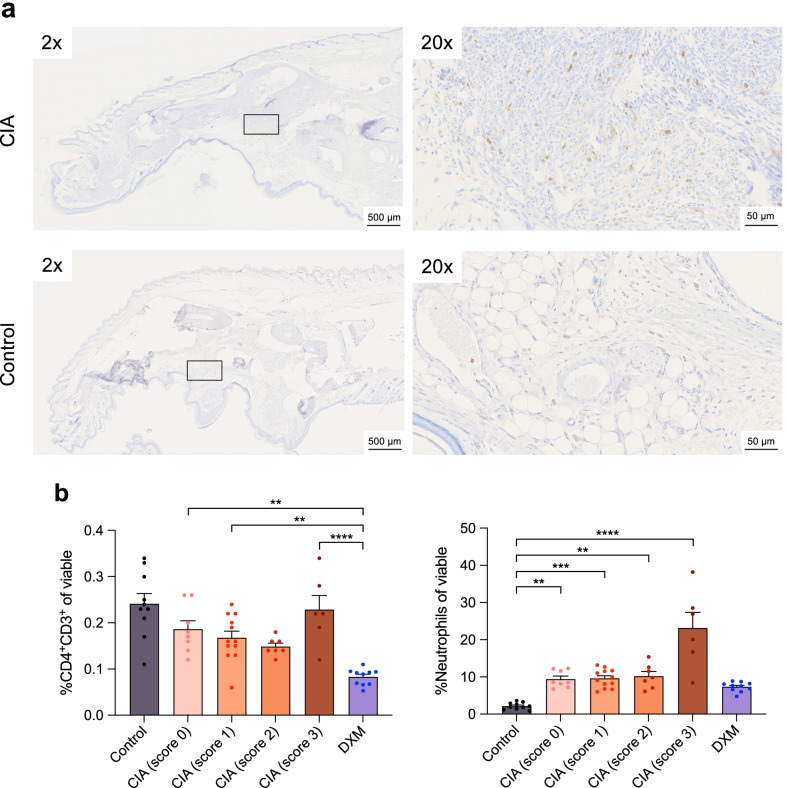
CD4^+^ T cell infiltration in arthritic joints. **a** Representative immunohistochemical images of CD4 infiltration in a joint with collagen-induced arthritis (CIA) (score 3; A: upper panels) and in a joint from a control mouse (A: lower panels). Mice with CIA were scored according to degree of inflammation (swelling and erythema) in each paw on a scale from 0 to 4. **b** Flow cytometric analysis with infiltration of CD4^+^ T cells and neutrophils as percent of viable cells in the carpal and tarsal joints from control, dexamethasone (DXM) treated and untreated CIA mice. Each point represents a joint. Gating strategy is shown in Additional file 1: Fig. S2. Data are presented as mean ± SEM and are pooled from two independent experiments with similar results. The significance level is indicated by asterisks (*). *p* < 0.01 (**), *p* < 0.001 (***) and *p* < 0.0001 (****)

To validate [^64^Cu]Cu-NOTA-CD4 tracer uptake, infiltration of CD4^+^ cells was evaluated in the joints by immunohistochemical analysis. IHC confirmed infiltration of CD4^+^ cells in arthritic joints compared with non-inflamed controls (Fig. 7a). Cell infiltration was generally increased in arthritic joints compared with controls. In addition, infiltration of CD4^+^ T cells and neutrophils in carpal and tarsal joints were evaluated by flow cytometry. Flow cytometric analysis revealed significantly increased CD4^+^ T cell infiltration in arthritic joints (score 3: 0.23 ± 0.03, *p* < 0.0001; score 2: 0.15 ± 0.01, *p* = 0.1187; score 1: 0.17 ± 0.01, *p* = 0.0046 and score 0: 0.19 ± 0.02% of viable cells, *p* = 0.0014) compared with DXM (0.08 ± 0.01%). Surprisingly, no significant differences between CIA and control mice were found as results revealed increased CD4^+^ cell infiltration (0.24 ± 0.02% of viable cells) in the joints of controls (Fig. 7b). However, significantly increased neutrophil infiltration was detected in CIA mice (score 3: 23.1 ± 4.2, *p* < 0.0001; score 2: 10.2 ± 1.2, *p* = 0.0018; score 1: 9.6 ± 0.7, *p* = 0.0008 and score 0: 9.4 ± 0.8% of viable cells, *p* = 0.004) compared with controls (2.2 ± 0.3% of viable cells). In addition, the neutrophil infiltration in joints with score 3 was also significantly increased compared with DXM (7.3 ± 0.4% of viable cells, *p* < 0.0001). Asterisks not shown on graph. Gating strategy, CD45^+^ T cells and viable cells in joints are shown in Additional file 1: Fig. S2.

## Discussion

In this study, we developed and evaluated a novel radiotracer, [^64^Cu]Cu-NOTA-CD4, for immunoPET imaging of murine CD4^+^ T cells. [^64^Cu]Cu-NOTA-CD4 was successfully synthesized by F(ab)’2 fragments of R-anti-mouse CD4 antibodies conjugated to a chelator and radiolabeled with copper-64. We found that [^64^Cu]Cu-NOTA-CD4 could be used for noninvasive visualization of CD4^+^ T cells. Importantly, [^64^Cu]Cu-NOTA-CD4 accumulates in T cell-rich tissues compared with the control isotype tracer, [^64^Cu]Cu-NOTA-IgG2b, and target-specific affinity was also supported by reduced uptake of [^64^Cu]Cu-NOTA-CD4 in T cell-depleted tissue.

Our findings showed that [^64^Cu]Cu-NOTA-CD4 accumulates in tissues with high level of CD4^+^ T cells and can follow stimuli affected T cell levels. Imaging of CD4^+^ T cells has an increasingly important role reflecting the pivotal role of CD4^+^ T cells in many inflammatory diseases including RA, inflammatory bowel disease and multiple sclerosis [6, 19, 20]. These conditions are CD4^+^ T cell driven, and CD4^+^ T cells play a crucial role in disease initiation. Previously, several monoclonal antibodies and their fragments have been radiolabeled and investigated for preclinical immunoPET utilization [9]. Researchers have especially taken advantage of immunoPET within the field of oncology for tumor associated antigens, but this has expanded to several indications including inflammation imaging [21].

In our studies, we used antibody F(ab)’2 fragments with two antigen-binding regions, as these have great potential as diagnostic components. Previous studies also demonstrated the use of radiolabeled antibody fragments for imaging of inflammation. Freise et *al.* developed a CD4^+^ T cell tracer of GK1.5 cys-diabody (cDb), an anti-mouse CD4 antibody fragment, radiolabeled with Zirconium-89 for PET imaging [22]. Here, [^89^Zr]Zr-malDFO-GK1.5 cDb detected CD4^+^ T cells in the distal colon of dextran sulfate sodium-induced colitis in a mouse model of inflammatory bowel disease. Beneficially, antibody fragments have shorter circulation times (hours), deeper tissue penetration, allow imaging the same day and can therefore be radiolabeled with shorter-lived PET radionuclides, e.g., flourine-18 (*t*_½_ = 109.7 min) and gallium-68 (*t*_½_ = 68 min). However, using positron emitters with a longer physical half-lives such as copper-64 (*t*_½_ = 12.7 h) enable immunoPET imaging the following day (24 h) [23]. In our longitudinal biodistribution study, the optimal imaging time point of 24 h p.i. was determined.

We also showed increased accumulation of [^64^Cu]Cu-NOTA-CD4 in the spleen and thymus compared with the isotype, [^64^Cu]Cu-NOTA-IgG2b. For further elucidation, splenic CD4^+^ T cell depletion was conducted by WBI in mice of 5 Gy. Accordingly, the uptake of [^64^Cu]Cu-NOTA-CD4 significantly decreased in irradiated mice compared with non-irradiated mice, supporting the target-specific binding of [^64^Cu]Cu-NOTA-CD4. Similar depletion has previously been shown by Wei et al. [24], who significantly reduced number of CD4^+^ T cells in the spleen two days after local tumor irradiation (8.5 Gy daily) of female C57BL/6JRj mice. Likewise, Li et al. [25] showed that 5 Gy of WBI significantly reduced the number of CD4^+^ T cells after seven days in the spleen. This clearly indicates that [^64^Cu]Cu-NOTA-CD4 targets CD4^+^ T cells but also clearly demonstrates the important differences between inflammatory and healthy tissues including vascular changes, increased blood flow, poor lymphatic drainage and edema [26].

Our findings in arthritic joints highlight an important challenge for large radiolabeled antigen-binding molecules, including the F(ab)´2 fragment. The results indicate that pathophysiological changes in lesions with pronounced inflammation stimulate tracer extravasation and retention. This may provide a situation where tracer activity and retention are the result of both target (ligand)-specific binding and an inflamed enhanced permeability and retention (EPR)-like effect to some degree. We have previously demonstrated that radiolabeled liposomes accumulate at very high levels in different types of inflammatory lesions [27]. This accumulation may illustrate the situation in cancerous tissues, where liposomes accumulate by the well-described EPR effect [28, 29]. The EPR effect results in non-specific extravasation of macromolecules from the blood stream via fenestration of the endothelial lining. The molecules are then retained from leaving the cancerous tissue [30]. In the light of the observed non-specific accumulation of radiolabeled F(ab)’2 fragments in severe inflammatory lesions, we therefore speculate that a comparable challenge may exist. In addition, we speculate that unspecific uptake of the tracer could be caused by increased phagocytic activity within the arthritic lesions with pronounced inflammation. This approach is also supported by the ex vivo analysis of the inflamed joints by flow cytometry, which showed increased number of neutrophils in joints with pronounced inflammation compared with healthy control and DXM-treated joints. Careful assessment of accumulation specificity and retention of F(ab)’2 fragments and comparable sized radiotracers are therefore warranted to secure validity.

Nevertheless, our results only revealed increased accumulation of isotype control, [^64^Cu]Cu-NOTA-IgG2b, in joints with pronounced arthritis. No increased uptake in tissues with mild arthritis and healthy tissue was observed, indicating specific targeting of our CD4 PET tracer, [^64^Cu]Cu-NOTA-CD4, in lesions with mild inflammation. Therefore, we speculate that the specificity of our CD4 PET tracer toward the increased number of CD4^+^ cells is sufficient to exceed the passive targeting by the EPR effect or potentially uptake by phagocytosis in joints with mild inflammation. Kristensen et al. [16] previously tested similar CD4^+^- and CD8^+^-specific PET tracers but radiolabeled with zirconium-89, [^89^Zr]Zr-DFO-CD4 and [^89^Zr]Zr-DFO-CD8a, in several preclinical mouse models of cancer. Here, tracers were used to phenotype tumors at an early stage and also allow following the treatment response. The possibility of unspecific tracer uptake in the heterogenic tumor microenvironment caused by the EPR effect was also discussed. Careful assessment and inclusion of isotype controls are therefore of great importance for indications beyond inflammation. Altogether, this indicates that the use of our CD4 PET tracer, [^64^Cu]Cu-NOTA-CD4, should be centered toward following the response to treatment and detecting potential recurrence rather than initial diagnosis. It could be argued that imaging of inflammation could easier be done with [^18^F]-FDG PET. However, it has previously been shown that not only is the uptake of [^18^F]-FDG low in arthritis but also that it seems not to correlate with severity of arthritis, i.e., arthritis score [31].

Radionuclide imaging of CD4 in arthritis adds to imaging of fibroblasts activating protein and F4/80 receptor positive macrophages [32].

## Conclusion

We developed and evaluated a novel radiotracer, [^64^Cu]Cu-NOTA-CD4, for immunoPET imaging of murine CD4^+^ T cells. [^64^Cu]Cu-NOTA-CD4 was successfully synthesized by F(ab)’2 fragments of R-anti-mouse CD4 antibodies conjugated to a chelator and radiolabeled with copper-64. We found that our novel CD4 PET tracer can be used for noninvasive visualization of murine CD4^+^ T cells and may be used for noninvasive studies of inflammatory conditions including RA.

## Supplementary Information

**Additional file 1: Figure S1** Full-length SDS-PAGE. (**a**) Coomassie staining of a full-length SDS-PAGE with ladder. (**b**) radiography of the same full-length stained SDS-PAGE. **Figure S2** Gating strategy used to identify CD4^+^ T cells and neutrophils by flow cytometry. Percent of (**a**) CD45^+^ T cells and (**b**) viable cells in the carpal and tarsal joints from control, DXM-treated and untreated CIA mice. Mice with CIA were scored according to degree of inflammation (swelling and erythema) in each paw on a scale from 0–4. Each point represents a joint. Data are pooled from two independent experiments. The significance level is indicated by asterisks (*). *p* = 0.02–0.04 (*) and *p* < 0.0001 (****). (**c**) Both populations were gated based on time, as singlets, based on scatter profile, lack of staining by viability dye and as positive for CD45. CD4^+^ T cells were further defined as CD3^+^ CD4^+^. Neutrophils were further defined as CD11b^+^ Ly6g^+^.

## Data Availability

The datasets generated during and/or analyzed during the current study are available from the corresponding author on reasonable request.

## Abbreviations

%Max ID/g: Maximum percent injected dose per gram
%ID/g: Percent injected dose per gram
%ID/joint: Percent injected dose per joint
^64^Cu: Copper-64
CD: Cluster of differentiation
CD4^+^ T cells: CD4^+^ T helper lymphocytes
CD8^+^ T cells: Cytotoxic CD8^+^ T lymphocytes
CIA: Collagen-induced arthritis
CII: Type II collagen
CT: Computed tomography
DXM: Dexamethasone
EPR: Enhanced permeability and retention
Gy: Gray
Ig: Immunoglobulin
IHC: Immunohistochemistry
i.p.: Intraperitoneal
i.v.: Intravenous
kDa: Kilodalton
MBq: Megabecquerel
MRI: Magnetic resonance imaging
NOTA: 1,4,7-Triazacyclononane-1,4,7-triacetic acid
PET: Positron emission tomography
p.i.: Post-injection
RA: Rheumatoid arthritis
ROI: Region of interest
SEM: Standard error of mean
WBI: Whole-body irradiation

## References

[1] Lee DM, Weinblatt ME (2001). Rheumatoid arthritis. Lancet.

[2] Pianta A, Arvikar SL, Strle K, Drouin EE, Wang Q, Costello CE (2017). Two rheumatoid arthritis-specific autoantigens correlate microbial immunity with autoimmune responses in joints. J Clin Invest.

[3] Tsark EC, Wang W, Teng YC, Arkfeld D, Dodge GR, Kovats S (2002). Differential MHC class II-mediated presentation of rheumatoid arthritis autoantigens by human dendritic cells and macrophages. J Immunol.

[4] Smolen JS, Aletaha D, Barton A, Burmester GR, Emery P, Firestein GS (2018). Rheumatoid arthritis. Nat Rev Dis Primers.

[5] Raza K, Falciani F, Curnow SJ, Ross EJ, Lee CY, Akbar AN (2005). Early rheumatoid arthritis is characterized by a distinct and transient synovial fluid cytokine profile of T cell and stromal cell origin. Arthritis Res Ther.

[6] Rao DA, Gurish MF, Marshall JL, Slowikowski K, Fonseka CY, Liu Y (2017). Pathologically expanded peripheral T helper cell subset drives B cells in rheumatoid arthritis. Nature.

[7] Aletaha D, Neogi T, Silman AJ, Funovits J, Felson DT, Bingham CO (2010). Rheumatoid arthritis classification criteria: an American College of Rheumatology/European League Against Rheumatism collaborative initiative. Arthritis Rheum.

[8] McInnes IB, Schett G (2011). The pathogenesis of rheumatoid arthritis. N Engl J Med.

[9] Signore A, Lauri C, Auletta S, Anzola K, Galli F, Casali M (2019). Immuno-imaging to predict treatment response in infection, inflammation and oncology. J Clin Med.

[10] Holmdahl R, Klareskog L, Rubin K, Bjork J, Smedegard G, Jonsson R (1986). Role of T lymphocytes in murine collagen induced arthritis. Agents Actions.

[11] Pietrosimone KM, Jin M, Poston B, Liu P (2015). Collagen-induced arthritis: a model for murine autoimmune arthritis. Bio Protoc..

[12] Laboratories H. Collagen-induced arthritis (CIA) in DBA/1 Mice. 2020.

[13] Kang I, Lee WW, Lee Y (2000). Modulation of collagen-induced arthritis by IL-4 and dexamethasone: the synergistic effect of IL-4 and dexamethasone on the resolution of CIA. Immunopharmacology.

[14] Oestergaard S, Rasmussen KE, Doyle N, Varela A, Chouinard L, Smith SY (2008). Evaluation of cartilage and bone degradation in a murine collagen antibody-induced arthritis model. Scand J Immunol.

[15] Scales HE, Ierna M, Smith KM, Ross K, Meiklejohn GR, Patterson-Kane JC (2016). Assessment of murine collagen-induced arthritis by longitudinal non-invasive duplexed molecular optical imaging. Rheumatology (Oxford).

[16] Kristensen LK, Frohlich C, Christensen C, Melander MC, Poulsen TT, Galler GR (2019). CD4(+) and CD8a(+) PET imaging predicts response to novel PD-1 checkpoint inhibitor: studies of Sym021 in syngeneic mouse cancer models. Theranostics.

[17] Lindmo T, Boven E, Cuttitta F, Fedorko J, Bunn PA (1984). Determination of the immunoreactive fraction of radiolabeled monoclonal antibodies by linear extrapolation to binding at infinite antigen excess. J Immunol Methods.

[18] Akitsu A, Ishigame H, Kakuta S, Chung SH, Ikeda S, Shimizu K (2015). IL-1 receptor antagonist-deficient mice develop autoimmune arthritis due to intrinsic activation of IL-17-producing CCR2(+)Vgamma6(+)gammadelta T cells. Nat Commun.

[19] Chitnis T (2007). The role of CD4 T cells in the pathogenesis of multiple sclerosis. Int Rev Neurobiol.

[20] Imam T, Park S, Kaplan MH, Olson MR (2018). Effector T helper cell subsets in inflammatory bowel diseases. Front Immunol.

[21] Reddy S, Robinson MK (2010). Immuno-positron emission tomography in cancer models. Semin Nucl Med.

[22] Freise AC, Zettlitz KA, Salazar FB, Lu X, Tavare R, Wu AM (2017). ImmunoPET imaging of murine CD4(+) T cells using anti-CD4 Cys-Diabody: effects of protein dose on T cell function and imaging. Mol Imaging Biol.

[23] Fu R, Carroll L, Yahioglu G, Aboagye EO, Miller PW (2018). Antibody fragment and affibody ImmunoPET imaging agents: radiolabelling strategies and applications. ChemMedChem.

[24] Wei S, Egenti MU, Teitz-Tennenbaum S, Zou W, Chang AE (2013). Effects of tumor irradiation on host T-regulatory cells and systemic immunity in the context of adoptive T-cell therapy in mice. J Immunother.

[25] Li X, Cui W, Hull L, Smith JT, Kiang JG, Xiao M (2018). Effects of low-to-moderate doses of gamma radiation on mouse hematopoietic system. Radiat Res.

[26] Medzhitov R (2008). Origin and physiological roles of inflammation. Nature.

[27] Clausen AS, Ostergaard DE, Holmberg P, Henriksen JR, Tham J, Damborg PP (2020). Quantitative determination of (64)Cu-liposome accumulation at inflammatory and infectious sites: potential for future theranostic system. J Control Release.

[28] Borresen B, Henriksen JR, Clergeaud G, Jorgensen JS, Melander F, Elema DR (2018). Theranostic imaging may vaccinate against the therapeutic benefit of long circulating PEGylated liposomes and change cargo pharmacokinetics. ACS Nano.

[29] Hansen AE, Petersen AL, Henriksen JR, Boerresen B, Rasmussen P, Elema DR (2015). Positron emission tomography based elucidation of the enhanced permeability and retention effect in dogs with cancer using copper-64 liposomes. ACS Nano.

[30] Xenaki KT, Oliveira S, van Bergen en Henegouwen PMP (2017). Antibody or antibody fragments: implications for molecular imaging and targeted therapy of solid tumors. Front Immunol.

[31] Terry SY, Koenders MI, Franssen GM, Nayak TK, Freimoser-Grundschober A, Klein C, Oyen WJ, Boerman OC, Laverman P (2016). Monitoring therapy response of experimental arthritis with radiolabeled tracers targeting fibroblasts, macrophages, or integrin αvβ3. J Nucl Med.

[32] Laverman P, van der Geest T, Terry SY, Gerrits D, Walgreen B, Helsen MM, Nayak TK, Freimoser-Grundschober A, Waldhauer I, Hosse RJ, Moessner E, Umana P, Klein C, Oyen WJ, Koenders MI, Boerman OC (2015). Immuno-PET and Immuno-SPECT of rheumatoid arthritis with radiolabeled anti-fibroblast activation protein antibody correlates with severity of arthritis. J Nucl Med.

