# Supplementary material for: Development of a 64Cu-labeled CD4+ T cell targeting PET tracer: evaluation of CD4 specificity and its potential use in collagen-induced arthritis
Source: EJNMMI Res. 2022 Sep 16;12:62. doi: 10.1186/s13550-022-00934-7 (PMC9481863; doi:10.1186/s13550-022-00934-7)
Supplement: Supplementary file 1 — Additional file 1: Figure S1 Full-length SDS-PAGE. (a) Coomassie staining of a full-length SDS-PAGE with ladder. (b) radiography of the same full-length stained SDS-PAGE. Figure S2 Gating strategy used to identify CD4+ T cells and neutrophils by flow cytometry. Percent of (a) CD45+ T cells and (b) viable cells in the carpal and tarsal joints from control, DXM-treated and untreated CIA mice. Mice with CIA were scored according to degree of inflammation (swelling and erythema) in each paw on a scale from 0–4. Each point represents a joint. Data are pooled from two independent experiments. The significance level is indicated by asterisks (*). p = 0.02–0.04 (*) and p < 0.0001 (****). (c) Both populations were gated based on time, as singlets, based on scatter profile, lack of staining by viability dye and as positive for CD45. CD4+ T cells were further defined as CD3+ CD4+. Neutrophils were further defined as CD11b+ Ly6g+. [file 13550_2022_934_MOESM1_ESM.docx]

**Supplementary material to**

**Title:**

Development of a ^64^Cu-labeled CD4^+^ T cell targeting PET tracer: evaluation of CD4 specificity and its potential use in collagen-induced arthritis

**Authors:**

Anne Skovsbo Clausen^1^, Camilla Christensen^1^, Esben Christensen^1,2^, Sigrid Cold^1,3^, Lotte Kellemann Kristensen^3^, Anders Elias Hansen^1,2^ and Andreas Kjaer^1,*^

**Author Information:**

^1^ Department of Clinical Physiology and Nuclear Medicine & Cluster for Molecular Imaging, Copenhagen University Hospital - Rigshospitalet & Department of Biomedical Sciences, University of Copenhagen, Blegdamsvej 9, DK-2100 Copenhagen, Denmark.

^2^ Department of Health Technology, Section for Biotherapeutic Engineering and Drug Targeting, Technical University of Denmark, Anker Engelunds Vej 1, DK-2800 Kgs. Lyngby, Denmark

^3^ Minerva Imaging, Lyshøjvej 21, DK-3650 Ølstykke, Denmark

**Corresponding Author (*):**

Professor Andreas Kjaer, MD, PhD, DMSc

Department of Clinical Physiology, Nuclear Medicine & PET and Cluster for Molecular Imaging, Department of Biomedical Sciences, Rigshospitalet and University of Copenhagen, Blegdamsvej 9, DK-2100 Copenhagen, Denmark. E-mail: [akjaer@sund.ku.dk](mailto:akjaer@sund.ku.dk)


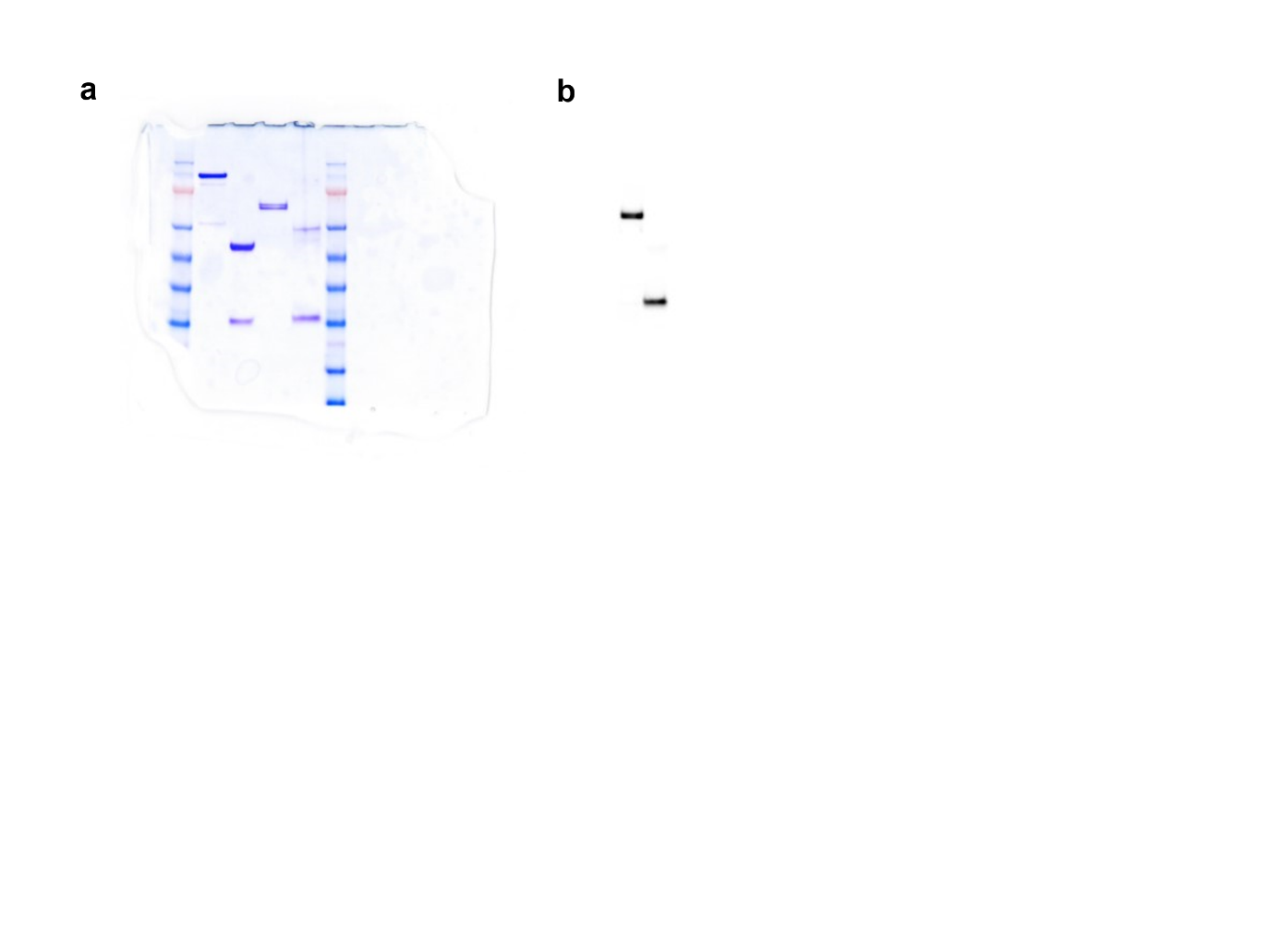
**Fig. S1.** Full-length SDS-page **(a)** Coomassie staining of a full-length SDS-page with ladder **(b)** radiography of the same full-length stained SDS-page. Exposure time 1 hour (read-out pixel size 25 µm, and read-out time 12 min)


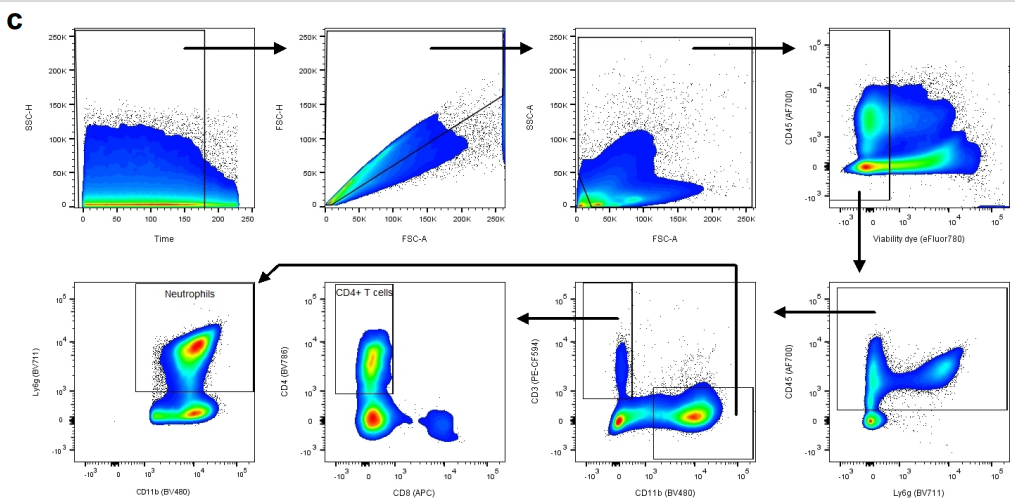

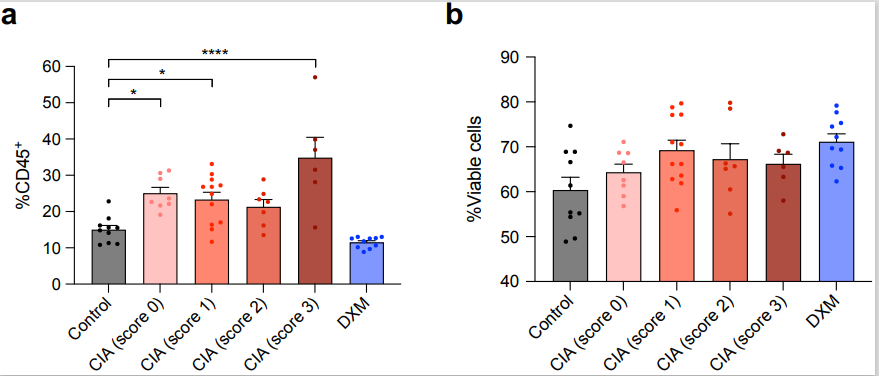
**Fig. S2.** Gating strategy used to identify CD4^+^ T cells and neutrophils by flow cytometry. Percent of (**a**) CD45^+^ T cells and (**b**) viable cells in the carpal and tarsal joints from control, DXM-treated and untreated CIA mice. Mice with CIA were scored according to degree of inflammation (swelling and erythema) in each paw on a scale from 0-4. Each point represents a joint. Data are pooled from two independent experiments. The significance level is indicated by asterisks (*). *p* = 0.02-0.04 (*) and *p* < 0.0001 (****). (**c**) Both populations were gated based on time, as singlets, based on scatter profile, lack of staining by viability dye and as positive for CD45. CD4^+^ T cells were further defined as CD3^+^ CD4^+^. Neutrophils were further defined as CD11b^+^ Ly6g^+^.
